# Genetic analyses of smoking initiation, persistence, quantity, and age-at-onset of regular cigarette use in Brazilian families: the Baependi Heart Study

**DOI:** 10.1186/1471-2350-13-9

**Published:** 2012-01-30

**Authors:** Andréa RVR Horimoto, Camila M Oliveira, Suely R Giolo, Júlia P Soler, Mariza de Andrade, José E Krieger, Alexandre C Pereira

**Affiliations:** 1 Laboratory of Genetics and Molecular Cardiology, Heart Institute, Medical School of University of São Paulo, Av. Dr. Enéas de Carvalho Aguiar, 44, São Paulo, SP, 05403-000, Brazil; 2 Department of Statistics, Polytechnic Center, Federal University of Paraná, Av. Cel. Francisco H. Santos, 100, Curitiba, PR, 81531-990, Brazil; 3 Department of Statistics, Mathematics and Statistics Institute, University of São Paulo, R. do Matão, 1010, São Paulo, SP, 05508-090, Brazil; 4 Department of Health Sciences Research, Mayo Clinic, 200 First Street S.W., Rochester, MN, 55905, USA

## Abstract

**Background:**

The purpose of this study was to estimate the genetic influences on the initiation of cigarette smoking, the persistence, quantity and age-at-onset of regular cigarette use in Brazilian families.

**Methods:**

The data set consisted of 1,694 individuals enrolled in the Baependi Heart Study. The heritability and the heterogeneity in genetic and environmental variance components by gender were estimated from variance components approaches, using the SOLAR (Sequential Oligogenic Linkage Analysis Routines) computer package. The mixed-effects Cox model was used for the genetic analysis of the age-at onset of regular cigarette use.

**Results:**

The heritability estimates were high (> 50%) for smoking initiation and were intermediate, ranging from 23.4 to 31.9%, for smoking persistence and quantity. Significant evidence for heterogeneity in variance components by gender was observed for smoking initiation and age-at-onset of regular cigarette use. Genetic factors play an important role in the interindividual variation of these phenotypes in females, while in males there is a predominant environmental component, which could be explained by greater social influences in the initiation of tobacco use.

**Conclusions:**

Significant heritabilities were observed in smoking phenotypes for both males and females from the Brazilian population. These data add to the literature and are concordant with the notion of significant biological determination in smoking behavior. Samples from the Baependi Heart Study may be valuable for the mapping of genetic loci that modulate this complex biological trait.

## Background

Annually, tobacco smoking is responsible for 5.4 million deaths worldwide [1], with more than 200,000 occurring in Brazil alone. Although smoking prevalence is decreasing as a result of public policies for the prevention and control of the tobacco epidemic, approximately 15.1% of Brazilian adults continue to smoke, with prevalence higher among males (17.9%) than females (12.7%) [2].

The natural history of addiction to nicotine can be characterized in stages. An individual first tries a puff or two, and eventually smokes a whole cigarette. Those who experience particular reinforcing biological or psycho-social influences will continue experimenting with smoking and may progress to regular use. With sufficient time and exposure, individuals with a set of predispositions and contextual influences will develop an addiction to nicotine [3]. Therefore, like drinking, smoking may be understood within a developmental framework determined by behavior, for which precursors are found in early childhood, and causal modifiers are evident throughout life [4].

Smoking behavior aggregates in families and in peer networks, due to genetic dispositions and familial and extra-familial environmental influences and it has been studied through several dimensions, such as smoking initiation, persistence and quantity. Although each one of these dimensions presents a particular genetic architecture, some genetic and environmental influences are shared between smoking persistence, smoking initiation [5,6], and nicotine dependence [7], suggesting an overlapping in the dimensionality of smoking behavior.

Previous studies have shown the heritability estimates for the different smoking dimensions to range from 21 to 84% [5,7,8] indicating a substantial genetic component. Significant gender differences were observed in the contribution of genetic and shared environmental effects to the total variance in these phenotypes [9,10].

In this paper, we estimate the genetic influences on the initiation, persistence, quantity and age-at-onset of regular cigarette use in the families of the Baependi Heart Study.

## Methods

### Study population and sample design

The Baependi Heart Study [11] is a genetic epidemiological study of cardiovascular disease risk factors, with a longitudinal design. Baseline enrollment occurred between December, 2005 and January, 2006, selecting 1,857 individuals distributed in 95 families resident in the municipality of Baependi, a city located in the Southeast of Brazil. Probands were identified from the community at large in several stages. Eleven census districts (from a total of twelve) were selected for study and the residential addresses within each district were randomly selected (first by randomly selecting a street, then randomly selecting a household). Only individuals age 18 and older, living in the selected household, were eligible to participate in the study.

Once a proband was enrolled, all his/her first-degree (parents, siblings, and offspring), second-degree (half-siblings, grandparents/grandchildren, uncles/aunts, nephews/nieces, and double cousins), and third-degree (first-cousins, great-uncles/great-aunts, and great-nephews/great-nieces) relatives and his/her respective spouse's relatives, who were at least 18 years old, were invited to participate. After the first contact with the proband, the first degree relatives were invited to participate by phone, including all living relatives in the city of Baependi (urban and rural areas) and surrounding cities. To recruit the participants, the study was advertised through provincial, religious, and municipal authorities, on local television, in newspapers and radio messages, through physicians, and by phone calls. For physical examination, a clinic was established in an easily accessible sector of Baependi.

Information regarding family relationships, sociodemographic characteristics, medical history, and environmental risk factors such as physical activity, smoking habit, and alcohol use were evaluated through a questionnaire completed by each participant. The questionnaire was based on the WHO-MONICA epidemiological instrument, and it was applied and filled out by research assistants specially trained for this task.

The study protocol was approved by the ethics committee of the Hospital das Clínicas, University of São Paulo, Brazil, and each subject provided informed written consent before participation.

### Measures

The smoking profile of this population was delineated through four dimensions: smoking initiation, persistence, quantity (related at average daily cigarette consumption), and age-at-onset of regular cigarette use. No other form of tobacco (pipe, cigar, etc.) was considered.

Five global questions collected the smoking data of the individuals. Two questions were related to smoking cessation and cigarette smoke exposure, but these data were not used in this study.

The first question assessed the smoking status of the individuals through three possible choices. "Did you already smoke cigarettes?" (1) Yes, in the past, but not currently; (2) Yes, and I still smoke; (3) I do not smoke. Option (2) refers to regular cigarette use, and the three choices are thus characterized as (1) former, (2) current, and (3) non- smokers, respectively. Individuals that never smoked, or who tried smoking a few times, but never smoked regularly were classified as non-smokers. The second question was related to the age-at-onset of regular smoking: "How old were you when you started smoking regularly?", and the last question assessed the average daily consumption of cigarettes: "How many cigarettes do you/did you use to smoke per day?" The two last questions were answered by former and current smokers.

### Statistical Analysis

Smoking initiation and persistence were analyzed as dichotomous variables, contrasting ever versus never, and former versus current smokers, respectively. The smoking quantity was analyzed as a continuous variable, representing the average number of cigarettes smoked per day. Natural log-transformation was applied for this trait in order to achieve the required normality assumption, enabling it to be analyzed as a continuous variable. The skewness and kurtosis statistics after natural log-transformation were - 0.39 and 2.31, respectively.

Familial correlations using the pairwise weighting scheme were computed for all main pair types of relatives available in the pedigrees employing the program FCOR of the computer package SAGE [12].

Polygenic heritability estimates for smoking initiation, persistence, and quantity were calculated using the variance-components approach contained in the SOLAR package [13]. In the variance component model, the level of the trait for individual *i *(denoted by γ*_i_*) is:

$$\gamma_{i}=\mu +\overset{c}{\underset{j=1}{\sum }}\beta_{j}{\text{X}}_{ij}+g_{i}+e_{i},$$

where μ is the general mean of the trait, and *β_j _*is the regression coefficient for covariate *j*, when applicable, which assumes the value *X_ij _*for individual *i*. The remaining parameters *g_i _*and *e_i _*are the residual genetic effect due the polygenic term, and random error component, respectively. The random effects *g_i _*and *e_i _*are assumed to be uncorrelated and normally distributed with mean zero and variance $\sigma_{g}^{2}$ and $\sigma_{e}^{2}$, respectively. As usual, the error component is unique to each individual, whereas the polygenic component is shared between individuals in proportion to their kinship coefficient. Thus, the covariance between traits for individuals *i *and *i' *is given by:

$$\left\{\begin{array}{c} \sigma_{g}^{2}+\sigma_{e}^{2},\;\text{for}\;\text{i}={\text{i}}^{\prime }\\ Cov\left(\gamma_{i},\gamma_{i}\right)=2\phi ii^{\prime }\sigma_{g}^{2},\;\text{for i}\neq {\text{i}}^{\prime },\text{but related}\\ 0,\text{for i}\neq {\text{i}}^{\prime },\text{but related} \end{array}\right)$$

The parameter 2*ϕii*' is the coefficient of the relationship between individuals *i *and *i'*. The likelihood of the traits of family members is assumed to follow a multivariate normal distribution. Estimates of the mean and variance components were obtained using maximum likelihood methods.

Two models were fitted to data of smoking initiation, persistence, and quantity, considering no covariate effects (model I), and age, sex, age^2^, and age by sex interactions effects, simultaneously (model II). In all analyses, the covariate age represents the age at the time of interview.

Household group analyses were also performed using the SOLAR system [13]. An additional variance parameter was added to model the effect of common environment, which is associated with any non-genetic factor shared between individuals living in the same household at the time of study. Using current residential addresses to define households, we have obtained 740 nuclear families from 95 families of the Baependi Heart Study. Household effects were investigated in both polygenic models (models I and II).

Models with distinct genetic and environmental variance components were also employed to evaluate the evidence of heterogeneity among genders in the heritability estimates of smoking initiation, persistence, and quantity, following the method described by Giolo et al. [14]. Assuming that the phenotypes in males and females are influenced by the same set of genes with distinct effects among genders, models I and II, described above, were fitted to the data. Again, the covariate age represents the age at the time of interview. Four situations regarding the genetic and environmental variance components among genders were considered: homogeneity in both variance components; heterogeneity in at least one of the variance components; heterogeneity only in the environmental variance components; heterogeneity only in the genetic variance components. Likelihood ratio tests were applied to define the models that presented the best fit to the data.

The variance-components approach is not appropriate for analyzing age-at-onset data due to the presence of censored observations. For age-at-onset of regular cigarette use, we used the random effects Cox proportional hazards model, proposed by Pankratz et al. (2005) [15], implemented in the *coxme *function of the R kinship library [16].

We fitted two mixed-effects proportional models to assess the genetic and shared family environmental factors influencing the age-at-onset of regular cigarette use. The first model corresponds to the polygenic effect shared by individuals within the family according to the degree of their relationships. The second model simultaneously includes both shared polygenic and shared family environmental effects. These same models were also employed to analyze the heterogeneity in variance components by gender. In all analyses, the covariate sex was included as a fixed effect in the model. Confidence intervals of the variance components were computed based on a profile-likelihood method [17]. This profile-likelihood method reduces the log-likelihood to a function of a single parameter of interest by treating the others as nuisance parameters and maximizing over them. A code in R [16] was used to obtain such confidence intervals.

It is not possible to obtain direct heritability estimates from mixed-effects Cox models, since there is no random error variance component. However, the variance components estimated may be interpreted as measures of familial aggregation [18]. The relative risk of the smoking behavior that corresponds to the random effect is obtained by exponentiation of the square root of the variance component. Relative risks for each covariate were obtained by exponentiation of their regression coefficients.

## Results

The smoking history of the families of the Baependi Heart Study was evaluated by the four described dimensions: initiation, persistence, quantity and age-at-onset of regular cigarette use. Of the 1,857 individuals selected in the baseline phase of the Baependi Heart Study, 1,694 (98.9%) answered the question that defines smoking initiation and persistent phenotypes, 648 (37.8%) the related question of daily cigarette consumption, and 635 (37.1%) indicated the age-of-onset of cigarette use. The data sets of smoking initiation and persistence were composed, respectively, of 1,282 individuals (1,010 non-smokers and 272 current smokers), and 683 individuals (411 former smokers and 272 current smokers). Non-smokers were not included in the quantity phenotype data set.

The sample characteristics are shown in Table 1. Among 1,694 adults participating in the study, 43.3% were male and 56.7% female, ranging in age from 18 to 95 years. The frequency of current smokers was 16%, and was higher among males (19.9%) than females (13.1%), and was in agreement with Brazilian indices. The frequency of young adult (18-24 years) current smokers (9.2%) was low compared to that of the Brazilian population (12.5%). Former smokers represent 24.2% of our sample, slightly higher than the average of the Brazilian population (22%) [2]. Current and former smokers smoked, on average, 14.1 cigarettes per day (range 1-80). The mean age-at-onset of regular cigarette use was 16.53 years, ranging from 7 to 50 years.

**Table 1:** Sample characteristics.

		Non-smokers		Former smokers		Current smokers		Overall
		
		%	n	%	n	%	n	n
Sex	male	34.5	349	58.2	239	53.7	146	734
	female	65.5	662	41.8	172	46.3	126	960

Age	18-24	20.0	202	5.6	23	9.2	25	250
	25-39	31.3	316	18.7	77	24.3	66	459
	40-54	22.6	229	44.1	181	41.2	112	522
	55+	26.1	264	31.6	130	25.3	69	463

Cigarettes smoked per day	1-10	NA	NA	51.3	211	51.8	141	352
	11-20	NA	NA	30.7	126	34.2	93	219
	21-30	NA	NA	4.9	20	5.1	14	34
	31+	NA	NA	10.2	42	0.4	1	43
	no answer	NA	NA	2.9	12	8.5	23	35

Age: age at the time of interview; NA: not available.

Familial correlations estimated in the various types of relatives are presented in Table 2. Significant non-zero correlations were observed among parent-offspring for all analyzed traits, among siblings for smoking initiation, and among mother-father pairs for smoking initiation and persistence. The results indicate that individuals with the closest relationships, genetically related or not, tend to be more similar in their smoking habits, mainly with respect to smoking initiation, suggesting that familial resemblance is probably associated with familial environmental factors shared by persons closely related.

**Table 2:** Interclass and intraclass correlations in different pairs of relatives for smoking initiation, persistence, and quantity.

Type of Relatives	Initiation Correlation (s.e.)	Persistence Correlation (s.e.)	Quantity Correlation (s.e.)
parent:offspring	0.09 (0.04)*	0.18 (0.07)*	0.16 (0.07)*
sibling	0.20 (0.04)***	0.07 (0.05)	0.12 (0.06)
half-sibling	-0.56 (0.25)	---	-0.55 (0.39)
grandparent	0.06 (0.08)	0.18 (0.17)	-0.08 (0.22)
avuncular	0.01 (0.04)	0.02 (0.05)	0.05 (0.06)
half-avuncular	-0.24 (0.28)	---	---
cousin	0.07 (0.04)	0.04 (0.05)	0.11 (0.07)
mother:father	0.24 (0.08)**	0.23 (0.11)*	-0.003 (0.123)

s.e.: standard error; * p ≤ 0.05 ** p ≤ 00; *** p ≤ 001.

Two polygenic models were fitted for smoking initiation, persistence, and quantity analysis, taking into account no covariate effects (model I), and age, sex, age^2^, and age by sex interactions effects. We did not observe significant household effects among these traits, independently of the model analyzed. The covariates that presented statistical significance (p < 0.001) in the polygenic model were retained in the model to evaluate the heterogeneity in variance components by gender.

The heritability estimates were high (> 50%) for smoking initiation and were intermediate, ranging from 23.4 to 31.9%, for smoking persistence and quantity. Although the adjustment of the models for covariates has improved the heritability estimates in all analyses, especially for smoking initiation, the covariates contributed with only a small proportion of the total phenotypic variance of each trait, ranging from 1.3 to 8.1% (Table 3).

**Table 3:** Heritability estimates for smoking initiation, persistence, and quantity in Brazilian families.

Smoking Dimension	Model	h^2 ^(s.e.), p value	$\sigma_{g}^{2}$	$\sigma_{e}^{2}$
Initiation	no covariate age, sex, age^2^	0.507 (0.106), p < 0.001 0.696 (0.123), p < 0.001	0.832 0.885	0.809 0.385

Persistence	no covariate age	0.285 (0.133), p = 0.0099 0.319 (0.145), p = 0.0066	7.859 6.870	19.676 14.665

Quantity	no covariate age, sex, age × sex, age^2^	0.234 (0.098), p = 0.0028 0.256 (0.098), p = 0.0012	0.227 0.238	0.745 0.692

h^2 ^(s.e.): heritability estimate (standard error); $\sigma_{\text{g}}^{2}$: polygenic variance estimate; $\sigma_{\text{e}}^{2}$: environmental variance estimate.

No significant gender differences were detected in the heritability of smoking persistence or quantity, regardless of the model employed. For smoking initiation, heterogeneity in both genetic and environmental variance components was observed for the model without covariate effects, with the heritability estimate for females (0.55) higher than that for males (0.47). These analysis results are shown in Table 4.

**Table 4:** Parameter estimates from heterogeneity analysis in variance-components by gender for smoking initiation.

Model	Heterogeneity in variance components by gender						Selected model
	h^2^_m_	h^2^_f_	σ^2^_g,m_	σ^2^_e,m_	σ^2^_g,f_	σ^2^_e,f_	Variance with heterogeneity
No covariate	0.475	0.547	1.559	1.721	0.519	0.430	genetic and environmental
age, sex, age^2^	0.696	0.696	0.885	0.385	0.885	0.385	no heterogeneity

h^2 ^(s.e.): heritability estimate (standard error); h^2^_m _: heritability estimate for males; h^2^_f _: heritability estimate for females; σ^2^_g,m _: polygenic variance estimate for males; σ^2^_e,m _: environmental variance estimate for males; σ^2^_g,f _: polygenic variance estimate for females; σ^2^_e,f _: environmental variance estimate for females.

In Table 5, we present the parameter estimates from Cox proportional hazards models for age-at-onset of regular cigarette use. There was no variation in the hazard ratios associated with covariate sex interaction among different models or analysis.

**Table 5:** Variance-components and hazard ratio estimates for age-at-onset of regular cigarette use in Brazilian families.

Model	No heterogeneity			Heterogeneity in variance components by gender			
	
	Hazard ratio	Variance components		Hazard ratio	Variance components		
	
	Sex	σ^2^_g_	σ^2^_f_	Sex	σ^2^_g,m_	σ^2^_g,f_	σ^2^_f_
Polygenic	0.43***	0.58		0.43***	0.21	0.40	
		(0.33-0.91)			(0.01-0.54)	(0.11-0.78)	

Polygenic and shared family	0.43***	0.37	0.17	0.42***	0.03	0.28	0.25
		(0.14-0.70)	(0.05-0.39)		(0.00-0.35)	(0.01-0.65)	(0.12-0.48)

***p ≤ 0.001; σ^2^_g_: polygenic variance estimate; σ^2^_g,m_: polygenic variance estimate for males; σ^2^_g,f_: polygenic variance estimate for females; σ^2^_f_: variance estimate for shared family effect.

In both polygenic and polygenic and shared family models, there was heterogeneity in genetic variance component by gender. From the polygenic model, we obtained an estimate of polygenic variance component equal to 0.21 for males, with a 95% confidence interval of 0.01 - 0.54. Considering that polygenic variance estimates can be interpreted as measures of familial aggregation [18], this result suggests an intermediate degree of familial aggregation associated with the age-at-onset of regular cigarette use and shows that the individual relative risk of cigarette use due to polygenic effects among males are on average exp(√0.21) = 1.58. Among females, we observed a moderate polygenic variance estimate of 0.40 (95% CI, 0.11 - 0.78) indicating a higher level of familial aggregation compared to males and a risk of cigarette use which may be 88% (exp(√0.40) = 1.88) higher than the average risk of this sample. To evaluate the contribution of each of these two components of unmeasured risk, we considered both polygenic and shared family environmental random effects simultaneously in the model. Under this model, the polygenic (for males and females) and shared family variance components were 0.03 (95% CI, 0.00 - 0.35), 0.28 (95% CI, 0.01 - 0.65), and 0.25 (95% CI, 0.12 - 0.48), respectively. Similar polygenic and shared family environmental factors influence the variation in the age-at-onset of regular cigarette use in females while environmental factors play a greater role than polygenic factors in males. Assuming additivity, total variance of males and females is $0.28\;\left(\sigma_{\text{g},\text{m}}^{2}=0.03+\sigma_{\text{f}}^{2}=0.25\right)$ and $0.53\;\left(\sigma_{\text{g},\text{m}}^{2}=0.28+\sigma_{\text{f}}^{2}=0.25\right)$, respectively. Therefore, males of families with smoking behavior may have relative risk of tobacco use 70% higher than the overall risk in the sample, while females may have approximately double the risk in relation to overall risk of this population.

## Discussion and Conclusions

This study investigates the smoking profile of a sample of Brazilian adults of the Baependi Heart Study using data collected by questionnaire based on the WHO-MONICA epidemiological instrument. We observed in our sample a frequency of former smokers slightly higher and a frequency of young adult (18-24 years) current smokers lower than the prevalence reported for the Brazilian population. This difference could be explained by the fact that Baependi is located in a rural area of Brazil. The frequency of current smokers in our sample (16%) is similar to Brazilian indices.

Several approaches have been considered to model the genetic contributions to smoking. While twin, family and adoption studies have been employed to dimension genetic factors, association, linkage, and animal studies have contributed to the identification of candidate genes and metabolic alterations involved in the determination of smoking phenotypes.

Different genomic regions have shown linkage with distinct smoking dimensions, suggesting that each one can be influenced by particular mechanistic pathways. Significant linkage signals were identified on chromosomes 6, 10 and 14 for smoking initiation; 2, 3, 10, 17, 20 and 22 for smoking quantity; 2 and 10 for nicotine dependence. Linkage to chromosomes 20 and 22 is to loci that reside near the alpha 4 nicotinic cholinergic receptor gene (*CHRNA4*) and to an intronic region of the *ADRBK2*gene, which encodes the beta-adrenergic receptor kinase 2, respectively. The *ADRBK2 *gene product is an interesting candidate protein for moderating nicotine dependence via regulating the reinforcing effects of catecholamines [8,19,20]. Other candidate genes involving in the neurobiology of smoking have been identified through genome-wide association studies. Single nucleotide polymorphisms at *BDNF*, *CYP2B6*, and *SLC6A3 *were associated with smoking initiation; at *GRPR*, *NR3C2 *with smoking persistence; at *CHRNA3*, *CHRNA5*, *MAOA*, *TRPV1*, *FOSB *and *EGLN2 *with smoking quantity; and at *SLC1A2*, *CYP2A6*, *RYR1*, and *CHRNA1 *with age at smoking initiation. *CYP2A6 *is a well-established candidate gene for smoking, which encodes an enzyme involved in the metabolic inactivation of nicotine to cotinine [21,22].

All analyzed traits showed a significant familial aggregation in this population. The heritability estimates for smoking initiation are high (0.51 from model I, and 0.70 from model II). Genetic influences on smoking initiation ranging from 0.11 to 0.75 [19,23-25] are reported in twin and sibling pair studies. In all cited studies, the smoking initiation phenotype was defined contrasting ever versus never smokers. Although our results are in accordance with reported values, the direct comparison of heritability estimates across studies is difficult because our estimates represent broad sense heritability while all cited studies deal with narrow sense heritability. Behavioral genetic research has defined smoking initiation as age of onset of regular smoking, age of initial experimentation (first puff on a cigarette), or lifetime ever/never smoking, but with varying requirements of what constitutes regular smoking: under some definitions, very infrequent smokers are classified along with those who have never initiated [4]. The criteria for ever smoker definition in our study were similar to other studies [19,25]. For smoking persistence, the heritability estimates in our sample are intermediate (0.28 from model I, and 0.32 from model II). Similar to smoking initiation, there are proxy measures for smoking persistence, such as number of cigarettes smoked per day, current tobacco use, or even nicotine dependence [4]. Heritability estimates reported for current smoking and nicotine dependence in twin samples are highest, ranging from 0.55 to 0.75 [24-27]. For smoking quantity, the heritability estimates in our sample are also intermediate (0.23 from model I, and 0.26 from model II) and lower than reported values of 0.51 in sibling pairs [19] and 0.57 in twin pairs [28]. Goode et al. (2003) [8] found an estimate of similar magnitude (0.21) for self-reported maximum number of cigarettes smoked per day from familial data, using variance components methods. In general, studies in the literature vary as to experimental design, methodology for parameter estimation, sample characteristics, and, mainly, in phenotype definition, making the direct comparison of the heritability estimates very difficult. Interestingly, no study similar to ours (i.e., extended family sampling) was found in the literature.

Genetic effects can be confounded by within-family transmission of behavioral patterns, thus household effects in the families of this population were investigated in all tested models. We found no evidence for significant household effects in the smoking initiation, persistence or quantity phenotypes. The significance of the household effect on smoking dimensions is inconsistent across previous studies. Shared environmental components obtained from twin samples account for about 20% of the variance of smoking initiation [7] and can be significant for both genders [27,29], in only one gender [24], or for different age groups [23]. Little [19,27,29-31] or no effect [24,26,32] from common environments have been reported for smoking persistence and quantity. These inconsistencies are not surprising as potential household effects may be heterogeneous and may represent unmeasured factors, such as family composition, neighborhood characteristics and cultural background. In addition, some studies report distinct genetic factors can be influencing the phenotypic expression of these traits in males and females [9,23,27,29]. Our results suggest genetic factors contribute differently to the phenotypic expression of smoking initiation in males and females; no evidence was found for distinct genetic factors influencing smoking persistence and quantity phenotypes among genders. The heterogeneity in results among the various studies indicates that knowledge about the genetic and environmental components of smoking variation is still incomplete.

A high degree of familial aggregation was also observed for age-at-onset of regular cigarette use, mainly in females, which presented relative risks 88 to 107% higher than the overall average risk for the entire sample. In males, there was a predominant environmental component, which could be explained by greater social influences in the initiation of tobacco use in this gender. The age-at-onset of tobacco use is strongly influenced by genetic factors, and differences in the risk of earlier smoking initiation have been observed between females and males [23,33]. Early age of onset of smoking is associated with heavier smoking as an adult, a reduced probability of successful smoking cessation, and an increased risk of early mortality [33]. Future studies, including the relation of the age-at-onset of regular cigarette use with socio-demographic characteristics, should be performed to better understand this phenotype in this population.

Our study presents some limitations. The assessment of the smoking behavior was obtained by self-report, and our findings can be subject to phenotypic misclassification, although this is a feature of other works on this topic. Furthermore, the data set used in this study was small in terms of subjects evaluated and controlled variables. The Baependi Heart Study is a longitudinal study and the first follow-up has been started. Besides the mapping of genetic loci, new approaches should be considered to better understand the smoking behavior in this population.

## Competing interests

The authors declare that they have no competing interests.

## Authors' contributions

ARVRH performed data analysis and interpretation, and wrote the manuscript. SRG, JPS, and MA participated in the concept and design of the study, and provided statistical support for data analysis and interpretation. CMO performed data acquisition and analysis. JEK participated in the concept and design of the study and was a manuscript reviser. ACP participated in the concept and design of the study, provided support for data analysis and interpretation, and was a manuscript reviser. All authors read and approved the final manuscript.

## Pre-publication history

The pre-publication history for this paper can be accessed here:

http://www.biomedcentral.com/1471-2350/13/9/prepub
